# Resolving trabecular metaphyseal bone profiles downstream of the growth plate adds value to bone histomorphometry in mouse models

**DOI:** 10.3389/fendo.2023.1158099

**Published:** 2023-03-30

**Authors:** P. L. Salmon, S. Monzem, B. Javaheri, L. Oste, G. Kerckhofs, A. A. Pitsillides

**Affiliations:** ^1^ Bruker Belgium (microCT), Preclinical Imaging, Kontich, Belgium; ^2^ Skeletal Biology Group, Comparative Biomedical Sciences, Royal Veterinary College, London, United Kingdom; ^3^ Galapagos NV, Discovery DMPK, Mechelen, Belgium; ^4^ Biomechanics Lab, Institute of Mechanics, Materials and Civil Engineering, Katholiek Universiteit van Leuven, Leuven, Belgium

**Keywords:** trabecular (cancellous) bone tissue, histomorphometry bone, growth plate, microCT (μCT), 3D imaging, osteoporosis, neurectomy, ovariectomy (OVX)

## Abstract

**Introduction:**

Histomorphometry of rodent metaphyseal trabecular bone, by histology or microCT, is generally restricted to the mature secondary spongiosa, excluding the primary spongiosa nearest the growth plate by imposing an ‘offset’. This analyses the bulk static properties of a defined segment of secondary spongiosa, usually regardless of proximity to the growth plate. Here we assess the value of trabecular morphometry that is spatially resolved according to the distance ‘downstream’ of—and thus time since formation at—the growth plate. Pursuant to this, we also investigate the validity of including mixed primary–secondary spongiosal trabecular bone, extending the analysed volume ‘upstream’ by reducing the offset. Both the addition of spatiotemporal resolution and the extension of the analysed volume have potential to enhance the sensitivity of detection of trabecular changes and to resolve changes occurring at different times and locations.

**Method:**

Two experimental mouse studies of trabecular bone are used as examples of different factors influencing metaphyseal trabecular bone: (1) ovariectomy (OVX) and pharmacological prevention of osteopenia and (2) limb disuse induced by sciatic neurectomy (SN). In a third study into offset rescaling, we also examine the relationship between age, tibia length, and primary spongiosal thickness.

**Results:**

Bone changes induced by either OVX or SN that were early or weak and marginal were more pronounced in the mixed primary–secondary upstream spongiosal region than in the downstream secondary spongiosa. A spatially resolved evaluation of the entire trabecular region found that significant differences between experimental and control bones remained undiminished either right up to or to within 100 μm from the growth plate. Intriguingly, our data revealed a remarkably linear downstream profile for fractal dimension in trabecular bone, arguing for an underlying homogeneity of the (re)modelling process throughout the entire metaphysis and against strict anatomical categorization into primary and secondary spongiosal regions. Finally, we find that a correlation between tibia length and primary spongiosal depth is well conserved except in very early and late life.

**Conclusions:**

These data indicate that the spatially resolved analysis of metaphyseal trabecular bone at different distances from the growth plate and/or times since formation adds a valuable dimension to histomorphometric analysis. They also question any rationale for rejecting primary spongiosal bone, in principle, from metaphyseal trabecular morphometry.

## Introduction

Rodents have been used for >50 years as models for skeletal biomedicine research. Their use often focuses on reproducing the osteopenic deterioration of trabecular bone architecture linked to sex hormone decline in ageing (1–3) and extends to the study of many factors that influence bone performance, including diet (4) and mechanical loading (5). Trabecular bone in the mouse distal femur or proximal tibia are the most commonly used sites in morphometric analysis both in 2D histological sections and increasingly in 3D micro-computed tomographic (microCT) images. Improving the methods that exploit these rodent trabecular sites would advance our scope to better understand bone pathobiology and likely speed the detection of new therapeutics for skeletal disorders.

**Figure 1:**
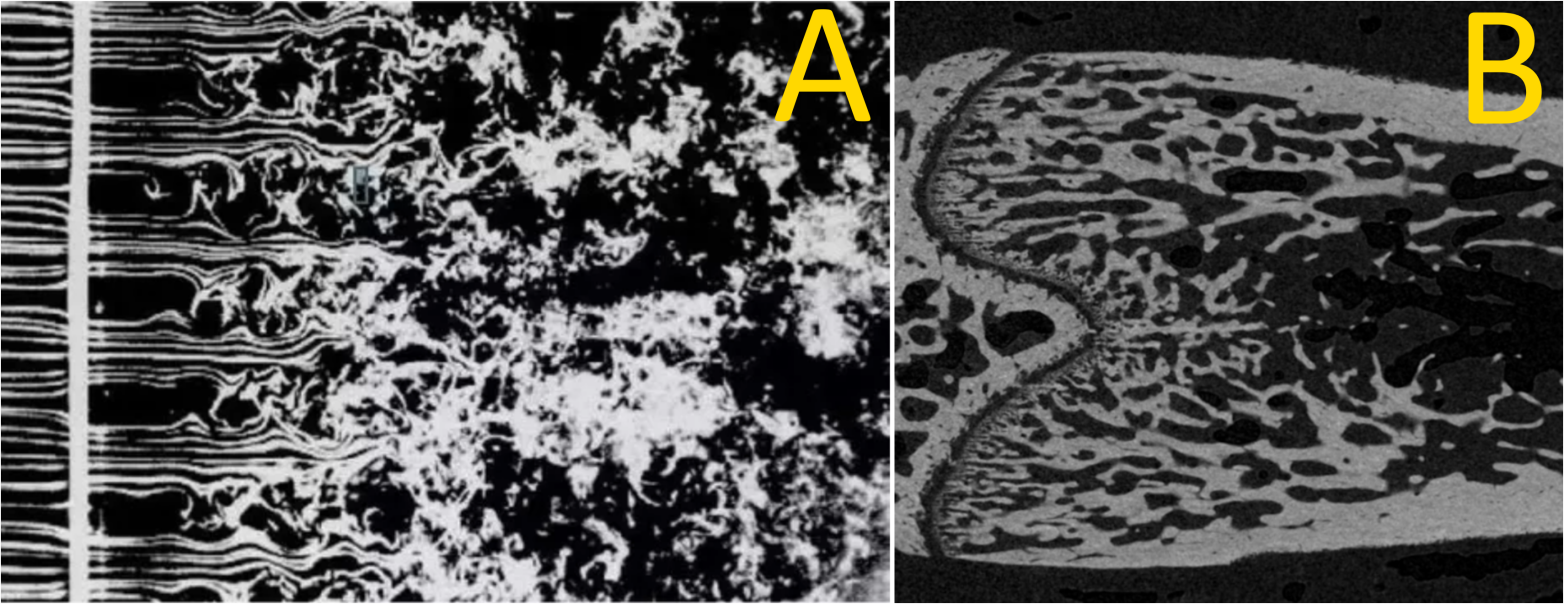
Smoke ‘wires’ in air passing through a grid **(A)** and a coronal section of a rat femoral metaphyseal trabecular bone downstream of the growth plate **(B)**. The similarity between these images points to similar dynamics: primary spongiosal pattern near the growth plate representing a laminar–turbulent transition with remodelling trabecular bone acting as a slow-flowing fluid. **(A)** by Thomas Corke and Hassan Nagib.

It is pertinent to first consider why rodent knee metaphyses have been favoured. One reason is pragmatic; they are rare sites in small rodents where trabecular bone is found in significant quantities. This is influential, as the study of trabecular architecture requires a minimum number, extent, and connectivity of its bony components and fortunate since trabeculae do not scale linearly with animal size (6, 7). Although the rodent knee metaphyses offer a large volume of trabecular bone, it is wrong to consider them as a uniform static bone compartment. During longitudinal bone growth, one can—in a relatively geometric sense—regard the trabecular spongiosa as ‘flowing’ away from the growth plate (*downstream* denotes its direction and conveys a dynamic quality; see **Figure 1**). In this regard, the standard method of trabecular morphometry currently imposes a delineation between the primary spongiosa, the fine and most recently created bone just downstream of the growth plate, and the secondary spongiosa, the more robust and wider-spaced bone further downstream (8). Here we explore a central tenet of trabecular morphometry, which is to place sole reliance on secondary spongiosa for analysis.

Traditionally, the selection of secondary spongiosa involves adding an ‘offset’ to some set distance downstream of the growth plate that serves to exclude the primary spongiosa. This, however, is complicated by the considerable and variable downstream extension of the primary spongiosa, its gradual merging into the secondary spongiosa that makes finding a clear boundary ambiguous, and potentially by the need for rescaling of the offset in mice of different ages, sizes, or sex. This offsetting may also omit significant secondary or include variable primary trabecular bone. The inference is that the primary spongiosa must be excluded from analysis as it displays a fundamentally divergent morphometric behaviour. The effect of offset size and whether morphological shifts in the primary and secondary spongiosa indeed diverge in response to pharmacological or biomechanical challenge have not been fully established by a morphometric study.

MicroCT-based 3D trabecular analysis is firmly based on 2D histomorphometric principles, retaining historical and well-used tenets that revolutionized bone analysis (1, 9). It was from such classic studies that the imposition of an offset originally emerged. Ke et al. (10) focused their measurements in 7-month-old rats on a central area starting 0.6 mm from the growth plate—the metaphyseal junction—rationalizing that this spotlighted the secondary spongiosa. Gallagher et al. (11) equally examined an area offset by 1 mm from the growth plate in rats, citing Wronski et al. (12), as a means of excluding the primary spongiosa. Offsets are also used in mice; a study on the effects of estrogen by Samuels et al. (13) applied a 0.25-mm offset as used before by Bain et al. (14). Although these studies did not justify these offset distances, there is a clear rationale. One reason is convenience: analysis is easier in the secondary spongiosa where the distinction from the cortical bone is simpler. Defining this offset distance is complicated, however, in studies involving rodents of different sizes, where the offset can require rescaling.

Another basis for selecting just the secondary spongiosa centres on (re)modelling kinetics and two traits that are absent in the primary spongiosa. First, modelling/remodelling of the secondary spongiosa occurs on a larger spatiotemporal scale and involves bone multicellular units, akin to adult human bone, which gives these analyses in rodents translational relevance. Second, the persistence argument: far downstream, spongiosa have been present for more of the experiment’s duration than newly formed primary spongiosa, giving secondary spongiosa more relevance. Few studies, however, have examined whether the expansion of the analysed volume to include mixed primary and secondary spongiosa modifies the recorded trabecular response to known challenges. Furthermore, it remains possible that, instead of a static bulk analysis of a defined trabecular volume, a spatially resolved metaphyseal trabecular bone analysis that adds the dimension of downstream distance (and hence time since synthesis at the growth plate) can enhance the knowledge gained of trabecular bone responses. Such spatially resolved analysis might identify conserved and also distinguish distinct sensitivities of bone responses across primary and secondary spongiosal regions.

**Figure 2:**
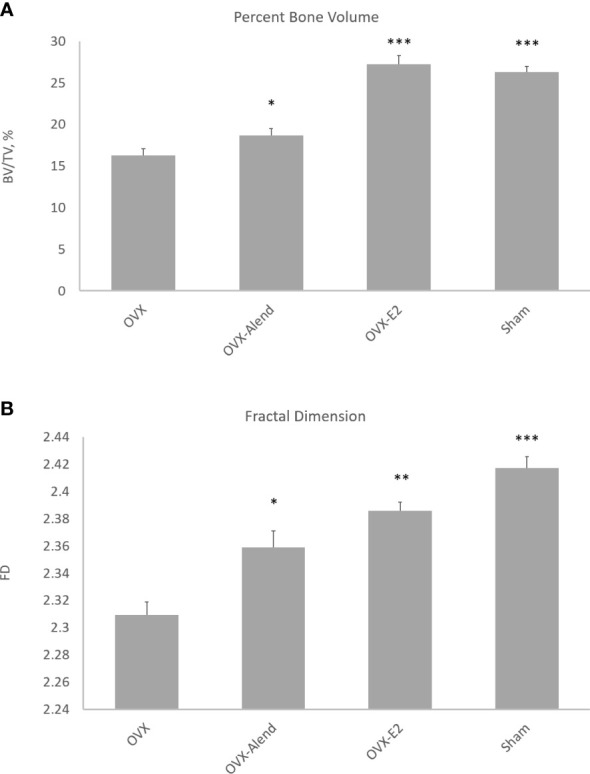
3D morphometry from study A of the whole metaphyseal region, with the primary and secondary regions combined. The effect of ovariectomy osteopenia on trabecular mass and architecture and the amelioration of this effect by alendronate and estradiol (E2) treatment are shown for the parameters percent trabecular volume **(A)** and fractal dimension **(B)**. *p < 0.05, **p < 0.01, ***p < 0.001.

Spatiotemporal evaluation of metaphyseal trabecular bone remodelling and growth has been done—for instance, the studies in rats by Kimmel and Jee (15) into bone-seeking radionuclides, where a meticulous histological analysis across multiple bands at specific distances downstream of the growth plate revealed two trabecular categories. A category that matched the primary spongiosa was <4.5 days old, with a high turnover and containing many osteoblasts and osteoprogenitor cells. The other—secondary spongiosa—had a relatively low turnover and less osteoblasts/osteoprogenitors. Likewise using similar methods, Fazzalari et al. (16) made a detailed spatially resolved morphometry, in human infant ribs, of cartilage within the growth plate and bone just downstream of it, providing downstream profiles of structural and cellular parameters. These authors afforded images of the trabecular region downstream of the growth plate (**Figure 2** in that paper) which made the analogy to a laminar-turbulent fluid flow transition (**Figure 1**) very compelling. The spatially resolved microCT analysis of rat distal femur trabecular bone by Gabet et al. in relation to orthopedic implants introduced the notion of a *metaphyseal gradient* of trabecular parameters (17), and we adopted this term.

Herein we examine two experimental studies which use micro-CT to describe spatially resolved architectural changes along the entire length of the metaphyseal trabecular compartment: in mice subjected to ovariectomy and supplementation with either estradiol or low-dose bisphosphonate (study A) and also in mice subjected to hindlimb disuse (study B). It is important to emphasise that these studies are not being presented primarily to report the results of these perturbations of the trabecular bone, which are well established and widely published. We evaluate instead the magnitude and statistical significance of the changes to the trabecular bone in relation to downstream distance and assess the value of adding this spatiotemporal dimension. The two studies are, to this end, deliberately chosen to include experimentally induced bone responses that are both stronger and weaker or of both earlier and later onset. We also test whether the offset applied near the growth plate can be shortened to include the primary spongiosa in the trabecular analysis. Another question addressed is if there are any morphological clues that pinpoint the primary-to-secondary spongiosa transition and any benefit accrued by making this distinction.

Finally, as the axial extent of the analysed region and the offset are both sometimes scaled to bone length (18), we also therefore, in study C, measure the tibia length and the primary spongiosal depth in the groups of mice from various experiments from early to late life to explore the validity of this simple scaling adjustment. How well do tibia length and primary spongiosal depth scale with each other and with age?

## Materials and methods

The microCT data from two studies in mice (studies A and B) were examined with the aim of resolving the differences in trabecular bone responses at different distances downstream of the growth plate in the femur and tibia, respectively. These studies employed either ovariectomy with or without exogenous hormone or pharmacological agent administration (study A) or neurectomy-induced disuse (Study B). A third study (C) employing data from several *in vivo* experiments compared the tibia length with the primary spongiosal depth and mouse age to assess mutual scaling among these three variables.

### Study A: Ovariectomy and osteopenia prevention by alendronate and estradiol

#### Animal procedures

C3H female mice were ovariectomized (OVX) or sham-operated under general anaesthesia by isofluorane at 10 weeks of age. The study was performed according to the ethical guidelines approved by the Animal Institutional Care and Use Committee of Galapagos NV controlled by the French Authorities (agreement number C93-063-06, DDPP, Seine Saint Denis). They were divided into four experimental groups, receiving drug or vehicle by daily oral gavage. The first group (sham) was sham-operated and treated with vehicle (*n* = 6). The second group (OVX) was OVX and treated with vehicle only (*n* = 6). The third group (alendronate) was OVX and treated with alendronate at a low dose of 1 mg/kg/day (*n* = 6). The fourth group (estradiol) was OVX and treated daily with 0.003 mg/kg/day estradiol (*n* = 6). The treatment duration was 5 weeks, commencing immediately after OVX or sham surgery, after which the mice were humanely euthanised. The right femurs were promptly harvested from all mice after euthanasia and fixed for 3 days in buffered formalin (1%), after which the bones were stored in 70% ethanol prior to microCT imaging.

#### MicroCT imaging

MicroCT imaging was performed using the SkyScan 1172 desktop scanner. The X-ray filter was 0.5 mm aluminium, with applied voltage of 50 kV and pixel size of 4.5 μm at intermediate CCD camera resolution with 2 × 2 pixel binning. The rotation step was 0.4°, and the scan orbit was 180° (plus cone angle). The camera exposure time was optimised, and the alignment and flat fields were up to date prior to scanning. The femurs were scanned mounted in plastic tube holders while wrapped in moist paper tissue for stability and to prevent desiccation during scans.

Raw projection files were reconstructed with a modified Feldkamp (19) algorithm using NRecon software (1.7.4.4). Beam hardening correction, Gaussian smoothing, and ring artifact reduction were applied. Attenuation contrast limits (intensity window) took a maximum value of approximately 15% above the highest bone attenuation and a minimum value of 5% of the maximum value but negative. With the exception of the post-alignment rotation axis correction for individual scans, the reconstruction settings were the same for all femurs. Following reconstruction, all femur 3D datasets were virtually reoriented in 3D using Dataviewer software (version 1.5) to ensure that the analysis was at the same location for each bone by consistent axial alignment. Each femur image was opened using this software, the 3D tri-axis view was loaded, and the tibia was reoriented in 3D by rotations in the transaxial, coronal, and sagittal planes and saved as new region of interest (ROI) datasets with the aim to standardize 3D orientation and crop out empty volume.

ROI selection, segmentation to binary images, and morphometric analysis were executed using SkyScan CT-Analyser (CTAn) software version 1.21.1.0. Volumes of interest (VOIs) for analysis were selected with reference to the growth plate. A cross-sectional slice was selected as the growth plate reference slice by finding the first visible bridging of the chondrocyte seam across the medulla while moving axially slice by slice upstream toward the growth plate from the metaphysis. A metaphyseal region was defined along the femur long axis commencing at this growth plate reference level and extending 2.25 mm (500 image slices) downstream. Within this metaphyseal VOI including both the trabecular and the cortical bones—the whole bone envelope—the separation of the trabecular bone from the cortical bone was done by an automated task list in CTAn using object labelling (despeckle) and morphological and Boolean operations. This task list started by inverse-binarising the medullary region followed by the removal of both trabecular structures and peripheral cortical-associated pores using the morphological escalator method (introduced herein) in which paired opening–closing operations are repeated with incrementally increasing diameter. Thresholding of greyscale images to binary was by global thresholding using a single greyscale and de-noising of binary images by a minor despeckle operation.

In the metaphyseal region of 500 cross-sections, the 100 slices nearest the growth plate were designated as the primary spongiosal region, based upon inspecting the architecture, and the remaining 400 slices as the secondary spongiosal region. The primary region comprised a mixture of primary and secondary spongiosal bone, while the primary spongiosa was absent from the secondary region.

3D/2D morphometric parameters were calculated for the selected trabecular VOIs. The 3D morphometric parameters were based on an analysis of a 3D model bounded by a surface rendered by the Marching Cubes method (20), and the 2D areas and perimeters were based on the Pratt (21) algorithm of sub-pixel perimeter interpolation. The morphometric parameters measured by CT-Analyser have been validated on both virtual objects and aluminium foil and wire phantoms (22). The trabecular thickness (Tb.Th) and separation (Tb.Sp) in 3D were both calculated using the local thickness or sphere-fitting (double distance transform) method (23), and the trabecular number (Tb.N) was calculated as 1/(Tb.Th + Tb.Sp) (this is not the default Tb.N output of CTAn but an alternative calculation). The Structure Model Index (SMI) was derived according to the method of Hildebrand and Ruegsegger (24) and the related parameter trabecular pattern factor (Tb.Pf) (25) was by a similar differential method based on surface dilation, as for SMI. The Un-Plate Index (uPi) was calculated as (BS × Tb.Th)/(2 × BV) (26). Euler connectivity (Conn.D) was calculated by Toriwaki and Yonekura’s algorithm (27) for simplicial decomposition of 3D cubic-voxelised objects. Fractal dimension (FD) was calculated in 3D by the Kolmogorov box counting method (28) over a box size range of 1–100 pixels (4.5–450 µm). The definitions, symbols, and units for bone morphometric parameters follow the ASBMR standardised nomenclature (29).

### Study B: Limb disuse by sciatic neurectomy

#### Animal procedures

Seventeen 12-week-old female C57BL/J6 mice were housed in polypropylene cages as groups of four to five and subjected to 12 h light/dark, with 19–23°C room temperature. They were fed *ad libitum* with a maintenance mice diet and water. All procedures complied with the Animals (Scientific Procedures) Act 1986 of the UK and with local ethics committee guidelines and were covered by HO Project license number 70/07859.

Each mouse was pre-medicated subcutaneously with 0.1 mg/kg buprenorphine (Vetergesic; Animalcare, York, UK), and anaesthesia was induced and maintained with isoflurane (Isoforine^®^-Cristália) diluted in 100% oxygen delivered by a mask. Sciatic neurectomy (SN) of the right limb of each mouse was accomplished as previously described, and the contralateral left limb served as an internal contralateral control (30). Briefly, an incision was made caudal to the right hip joint, and the biceps femoris muscle was elevated to expose the nerve. SN was achieved by resecting a 3- to 4-mm segment of the sciatic nerve posterior to the hip joint. The neurectomised mice were able to move around in the cage and gained access to food and water without difficulty. The mice were sacrificed through cervical dislocation at one of three different timepoints: either 5 days (*n* = 5), 35 days (*n* = 4), or 65 days (*n* = 5) after right SN. The tibias, both left and right, were subsequently dissected and fixed in neutral-buffered formaldehyde for 24 h before washing and storage in 70% alcohol prior to microCT scanning.

#### MicroCT imaging

To enable the effects of different durations of disuse on bone architecture, mass, and shape to be evaluated, the left and right tibias of each mouse were scanned using SkyScan 1172 (Bruker microCT, Kontich, Belgium) with the following setup: 0.5 mm aluminium filter, 5 µm voxel size, 50 kV applied voltage, and intermediate camera resolution with 2 × 2 pixel binning (integration) at acquisition. The scan orbit was 360°. The right and left tibiae were scanned mounted in plastic tube holders while wrapped in moist paper tissue for stability and to prevent desiccation during scans.

The reconstruction and analysis of microCT images for study B were the same as for study A, except that, starting from the growth plate reference level and moving downstream, 400 instead of 500 cross-section slices were analysed. Within these 400 cross-sections, the 50 slices nearest the growth plate were designated (by visual inspection) as the primary spongiosa and the remaining 350 slices as the secondary spongiosal region. As in study A, the ‘primary spongiosal region’ comprised a primary/secondary spongiosal bone mixture, while a primary spongiosa was absent from the ‘secondary’ region.

#### Analysis of spatially resolved trabecular bone responses

The goal of this analysis in both studies A and B was to compare changes in the trabecular bone response with distance downstream of the growth plate. This was achieved by analysing the trabecular morphometry in three ways. First, the results are presented in an overview of the quantitative 3D morphometric analysis of the trabecular bone in the whole metaphyseal region combining the primary and secondary spongiosa. Secondly, a 3D morphometry of the metaphyseal trabecular bone was completed separately for the primary region nearest the growth plate and the secondary spongiosal region further downstream. (The locations and the extents of the primary and secondary regions in the femurs of study A and the tibias of study B are as described above.) In both studies A and B, the primary region corresponds to the region usually excluded in histomorphometry by the offset.

Thirdly, bone morphometry was carried out in 2D, cross-section by cross-section, downstream from the growth plate reference level through the whole metaphysis to provide a spatially resolved metaphyseal profile of the measured 2D parameters with increasing distance from the growth plate. At every cross-section at every distance downstream, analyses were performed by R software, version 4.0.2, using chi-square test to assess the overall effects and the significance of treatment among OVX, sham, estradiol, and alendronate groups in paired inter-group comparisons (study A) or between neurectomised and contralateral control tibia pairs (study B). For clarity, the statistical significance of differences with distance from the growth plate is presented as coloured bars accompanying the metaphyseal profiles—with error bars—of cross-sectional morphometric parameters.

### Study C: Comparison of primary spongiosal depth with tibia length and mouse age

In study C, the tibias were harvested post-mortem from control mice, which were not subjected to experimental interventions, at ages from 3 to 86 weeks (*n* = 3 to 4) from several *in vivo* studies conducted at the Royal Veterinary College, London, investigating the effects of either drug treatment or procedures such as neurectomy or mechanical loading on mouse tibia. The study methodology including animal ethics approval, care and handling, and microCT imaging followed the methods described for study B. The tibias were microCT scanned in their entirety by multi-part ‘oversize’ scans.

The tibial length and the depth of the growth plate were measured in whole microCT images. For the tibial length, the proximal landmark was defined as the cross-section level where the tibial condyles separated in articulation with the femur, and distal landmark was likewise where the tibial condyles at the ankle separated in articulation with the tallus (see (31)). The depth of the primary spongiosa was defined as the number of cross-sections between the growth plate reference level as described for study A and the visually identified ending of the main primary spongiosal structures—multiplied by the cross-section thickness of one (isotropic) voxel.

## Results

### Study A: Unified morphometry of the whole spongiosa discloses differing protection against ovariectomy-induced bone loss by estradiol and low-dose alendronate

The 3D morphometry over the whole metaphyseal region showed that OVX produced very marked and significant reductions in trabecular mass and alterations in organisation. Five weeks of exogenous estradiol treatment was fully effective, but low-dose alendronate was only marginally effective, in preventing OVX-induced osteopenia (**Figure 2**). The expected OVX-induced reduction in percent trabecular volume (BV/TV) was ameliorated by both alendronate (*p* < 0.05) and estradiol (*p* < 0.001) administration (**Figure 2A**), albeit with a higher significance from estradiol. The weaker more marginal effect of alendronate could prove useful, allowing evaluation of the sensitivity of morphometric analysis to small trabecular bone responses across the different metaphyseal spongiosal regions.

FD was also significantly reduced by OVX, and this was ameliorated by both alendronate (*p* < 0.05) and estradiol (*p* < 0.01) treatment (**Figure 2B**)—again with a higher significance for the latter drug. FD is employed here as a general index of complexity; it is also important as a signature of nonlinear pattern formation, the underlying process behind the emergence of chaotic labyrinthine trabecular bone architecture (32, 33).

### Study A: Separate 3D analysis discloses conserved primary and secondary spongiosal sensitivity to ovariectomy and to exogenous estradiol and alendronate

**Figure 3:**
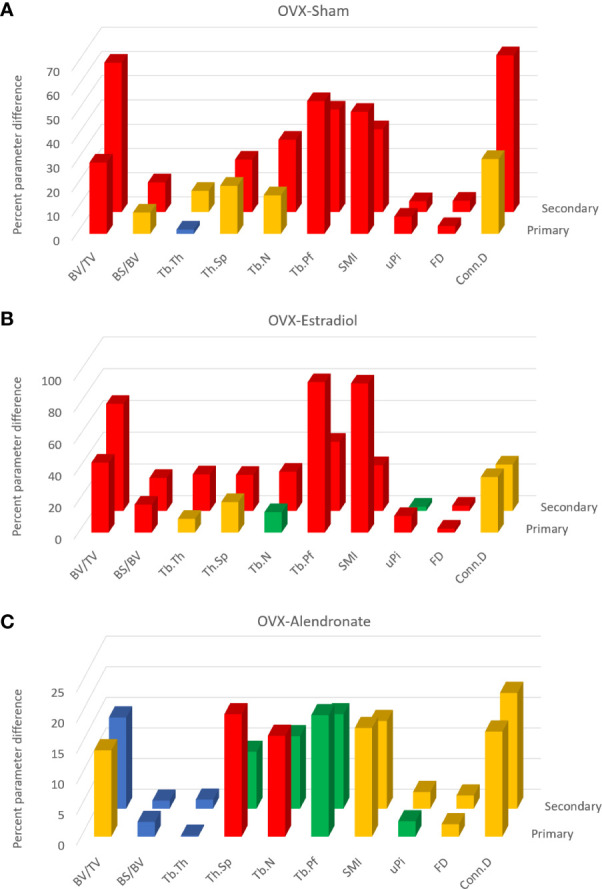
From study A, histograms of the trabecular bone parameter differences with paired-group comparisons: ovariectomy (OVX)–sham **(A)**, OVX–estradiol **(B)**, and OVX–alendronate **(C)**. The two rows of 3D columns show the data for the primary (front) and secondary (rear) spongiosal regions as labelled. ‘Primary’ refers to the 100 slices closest to the growth plate, while ‘secondary’ refers to the subsequent 400 slices from cross-section number 100 to 500 downstream. Note that the vertical *y* axis denotes not the parameter values *per se* but the percent difference thereof (made positive) between the paired groups. Colour coding of the parameter bars indicates statistical significance of difference according to Student’s *T*-test (two-tailed, heteroscedastic). Blue, *p* > 0.05; green, *p* < 0.05, *p* > 0.01; orange, *p* < 0.01, *p* > 0.001; red, *p* < 0.001.

Data from study A were also subjected to a separate 3D evaluation of primary and secondary regions (**Figures 3A–C**). Please note that these figures do not show parameter values but instead show for each parameter the OVX-induced differences relative to sham (**Figure 3A**), by estradiol relative to OVX (**Figure 3B**) and alendronate treatment relative to OVX (**Figure 3C**), as a positive percentage difference independent of the direction of change. Furthermore, the histogram bars in **Figure 3** are colour-coded to the significance of difference.

Comparing firstly the sham and OVX-vehicle groups, almost all parameters exhibited a highly significant response (% change) to OVX in both primary and secondary regions (**Figure 3A**), except Tb.Th in the primary region. The magnitude of these changes for the parameters of bone quantity (BV/TV, Tb.N) was greater in the secondary region, while for some parameters of the trabecular architecture (Tb.Pf, SMI, and uPi) the changes were greater in the primary region. The statistical significance of differences was somewhat less marked in the primary region than in the secondary spongiosal region (*e*.*g*., for BS/BV, Tb.Sp, Tb.N, and Conn.D).

The comparison across OVX-vehicle and estradiol-treated groups (**Figure 3B**) revealed that estradiol exerted a marked effect on all trabecular parameters, mostly with a very high level of significance in the primary region as well as in the secondary region. Whilst attaining a statistically significant change in the primary region, some parameters such as Tb.Th, Tb.Sp, and Tb.N nevertheless exhibited greater magnitude and significance of change in response to estradiol in the secondary spongiosal region (**Figure 2B**). BV/TV showed a lower change in the primary region, while again several parameters indicative of architecture rather than quantity (SMI, Tb.Pf and uPi) showed, conversely, more marked differences in the primary region than in the secondary metaphyseal region.

In contrast, low-dose alendronate only partially protected against the trabecular changes induced by OVX (**Figure 3C**). Notably, BV/TV was significantly increased in the primary region but not in the secondary region. For nearly all parameters, the differences induced by alendronate were always at least as marked, both statistically and in magnitude, in the primary spongiosa as in the secondary spongiosa (**Figure 3C**). As for BV/TV, the significance of changes in Tb.Sp and Tb.N due to alendronate treatment were greater in the primary spongiosa than in the secondary spongiosa (**Figure 3C**), although the change in uPi was less significant.

### Study A: Spatially resolved metaphyseal profiling reveals no general loss of detection of a trabecular response in the primary spongiosa

**Figure 4:**
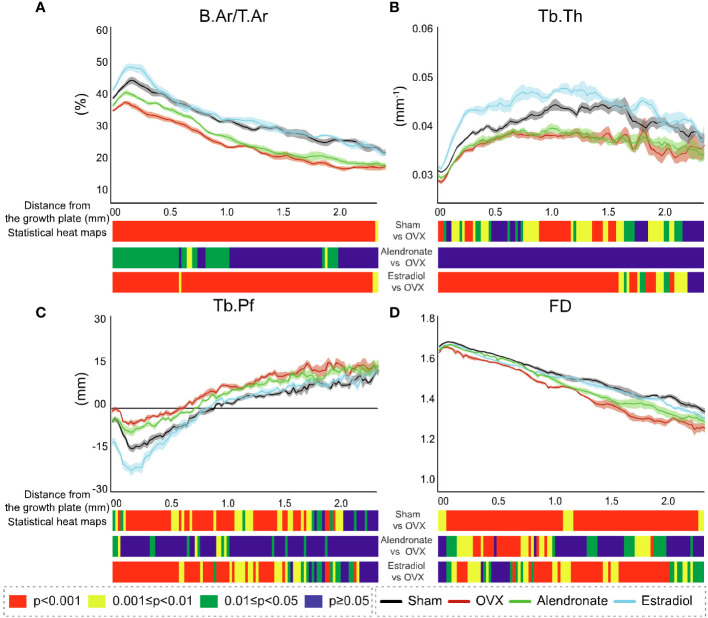
Profiles of the cross-sectional morphometric parameters in 2D with distance downstream of the growth plate from study **(A)**. Corresponding profiles are shown for each of the four groups: sham (black line), ovariectomy (red line), alendronate (green line), and estradiol (light blue line) in B.Ar/T.Ar **(A)**, Tb.Th **(B)**, Tb.Pf **(C)**, and FD **(D)**. For all profiles, the SEM ranges are given. Accompanying each graph is a composite colour bar indicating, for the three inter-group paired comparisons, the slice-by-slice *p*-value of the significance of difference (by chi-square), again in axial profile with distance downstream of the growth plate.

To probe the scope for divergent sensitivity in primary and secondary bone, we performed cross-sectional slice-by-slice profiling over the full metaphyseal VOI with a view to spatially resolving the 2D morphological changes with a specific distance from the growth plate. Such profiling is shown for B.Ar/T.Ar (percent bone area per total area), Tb.Th, Tb.Pf, and FD (**Figures 4A–D**, respectively), accompanied by statistical colour ‘heat’ maps with location-matched 2D *p*-values from chi-square analysis for the three comparisons (OVX–sham, OVX–estradiol, and OVX–alendronate). This profiling demonstrated increasing B.Ar/T.Ar immediately downstream of the growth plate that attained peak values within only a short distance (100–200 um) and thereafter an inflection to an expected quasi-linear downstream decline in all groups (**Figure 4A**). A close examination of the B.Ar/T.Ar profiles near the growth plate shows some informative divergences between groups. The “upslope” from the growth plate to the maximum attained peak, before the subsequent downslope at all distances further downstream, and the downstream distance of this peak probably indicate the depth of the primary spongiosa. If this is so, then OVX appears to reduce the primary spongiosal depth by almost half, and while the alendronate treatment does not seem to change the reduced depth of primary spongiosa (as indicated by the downstream distance of the B.Ar/T.Ar peak), the estradiol treatment almost fully restores its apparent depth to the sham value. Furthermore, while all three B.Ar/T.Ar profiles—except for the downstream distance of the maximum—were approximately parallel to each other, the profile for the estradiol treatment group shows an exceptional upward excursion from the growth plate to approximately 300 µm downstream, after which it closely tracks the sham group profile.

The location-matched 2D *p*-values revealed a marked decline in B.Ar/T.Ar in response to OVX (**Figure 4A**), with high levels of significance along the entire profile spanning primary and secondary regions. Estradiol completely reversed these OVX-induced declines in B.Ar/T.Ar across the entire profile with high significance. In contrast, low-dose alendronate produced a significant reversal in B.Ar/T.Ar loss only in regions that extended to ~1 mm downstream, with no consistent increase evident further downstream—that is, a stronger response in the primary region than in the secondary region.

The profiling of Tb.Th (**Figure 4B**) revealed a minimum thickness in the fine-textured primary spongiosa which increased to a mid-metaphyseal maximum at ~1–1.5 mm downstream, with low values as expected toward the metaphyseal terminus. The location-matched comparison for Tb.Th in response to OVX showed a significant change with a variable significance up to 2 mm downstream, while in response to estradiol, the changes showed a stronger response over the same 2-mm downstream extent with mostly continuous high significance. However, there was no significant modification in Tb.Th exerted by alendronate treatment anywhere along the metaphyseal profile.

The profiling showed a very different trend for Tb.Pf (**Figure 4C**), with a decline to negative values just downstream of the growth plate followed by a positive (upward) inflection in values at ~0.1–0.2 mm, followed by a continuous increase further downstream in all groups. An examination of *p*-values showed statistically significant changes in TB.Pf in response to both OVX and estradiol up to approximately 2 mm downstream with the significance generally increasing with the proximity to the growth plate. The alendronate treatment, by contrast, generated limited intermittent increases in Tb.Pf that were again restricted to the region ~1 mm downstream of the growth plate.

The values for FD (**Figure 4D**) showed a remarkably linear decline along the metaphyseal profile in all groups, with a small exception at the growth plate. A highly significant decline in FD was found along the entire metaphyseal profile in response to OVX. The FD changes induced by estradiol that were protective against the effect of OVX also reached a high significance but were more intermittent over the metaphysis, with (in contrast to other parameters) a less significant change close to the growth plate. Notably, the cross-sectionally mapped changes in FD induced by alendronate (while weaker than those from OVX and estradiol) were much more significant in this group than for any other parameter and most significantly targeted trabecular regions ~0.2–0.9 mm downstream of the growth plate.

These data show the widespread impact of OVX on the metaphyseal profiles of all four parameters, with downstream declines in impact evident only for Tb.Pf and Tb.Th. Estradiol targets near-identical locations to OVX, with an especially strong effect on Tb.Th as is known from the bone anabolic effect of this hormone at a sufficient dose (34). An exception is that FD is less modified near the growth plate by estradiol than by OVX. In contrast, the locations modified by low-dose alendronate are always closer to the growth plate especially for B.Ar/T.Ar and FD. In general, the responses in the metaphyseal profiles between the paired groups were at least as strong in the primary spongiosal region as in the secondary spongiosal region.

### Study B: Unified morphometry of the whole spongiosa shows a prolonged, time-dependent neurectomy-induced decline in trabecular volume and organisation

**Figure 5:**
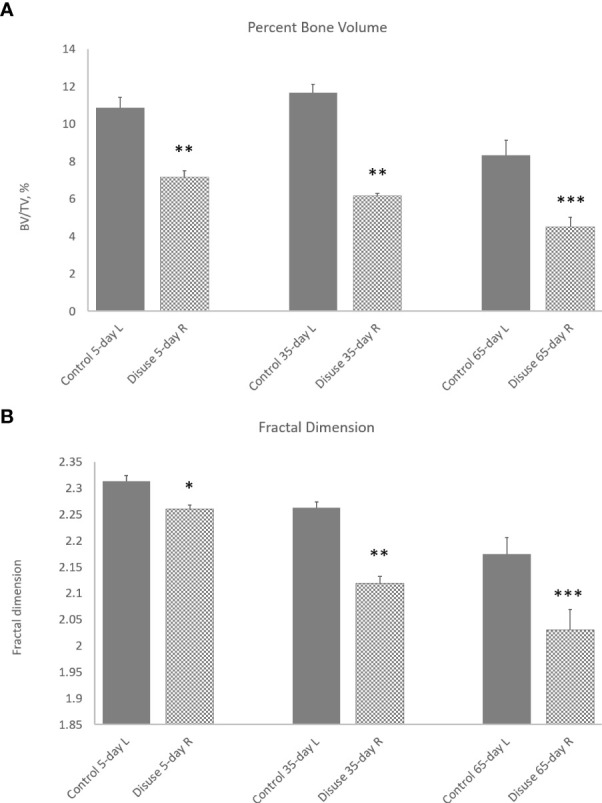
Effect of neurectomy-induced disuse on **(A)** percent trabecular bone volume (BV/TV) and **(B)** trabecular fractal dimension (FD) measured in 3D over the whole metaphyseal region (primary and secondary combined) at 5, 35, and 65 days post-operation. Grey bars, control left tibias; hatched bars, SN right tibias. *p < 0.05, **p < 0.01, ***p < 0.001.

The morphometric 3D evaluation of all metaphyseal trabecular bone in an overview disclosed a rapid SN-induced decline in BV/TV by 30% in 5 days (*p* < 0.01), which progressed to a greater decline at 35 and 65 days (*p* < 0.01 and *p* < 0.001, respectively; **Figure 5A**). FD also showed a similar decline trajectory within 5 days (*p* < 0.05), which progressed to larger decreases after 35 and 65 days (*p* < 0.01 and *p* < 0.001, respectively; **Figure 5B**). Note the non-zero origin for FD in **Figure 5B** and also that very small percent changes in FD can nonetheless have significance.

### Study B: Separate 3D analysis discloses distinct trajectories of neurectomy-related modification in primary and secondary spongiosa

**Figure 6:**
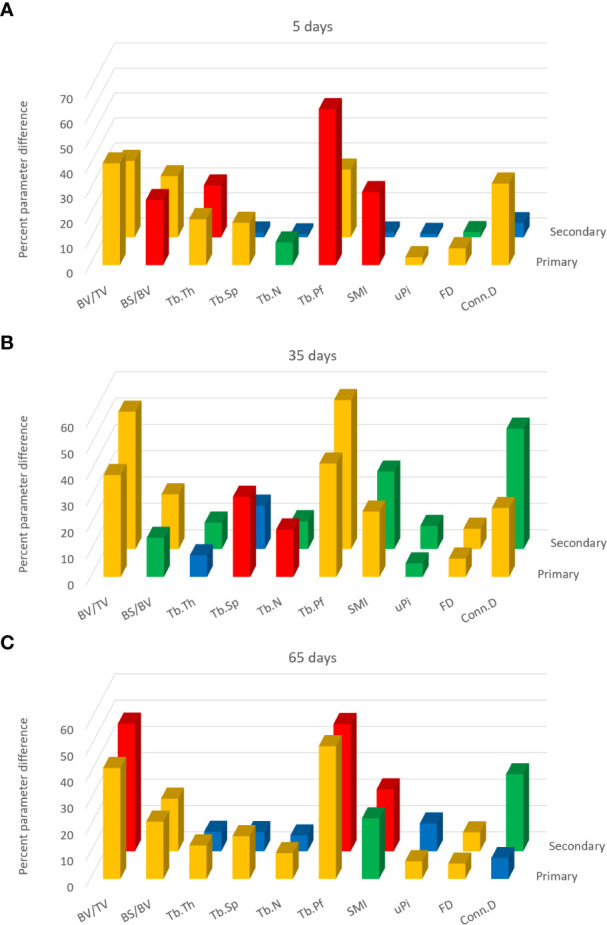
3D analysis of response to sciatic neurectomy (SN) in primary and secondary spongiosal trabecular regions for the **(A)** 5-, **(B)** 35-, and **(C)** 65-day post-neurectomy groups. These charts do not show the trabecular parameter values *per se* but the percent differences between the neurectomised right and control left tibias (made positive) for each parameter. The two rows of bars denote the primary spongiosa in front and the secondary spongiosal region behind. Colour coding of the bars is according to the *p*-values of the SN-contralateral differences from the Student’s *T*-test (paired, heteroscedastic). Blue: *p* > 0.05; green: *p* < 0.05, *p* > 0.01; orange: *p* < 0.01, *p* > 0.001; red: *p* < 0.001.

The data from study B were also separately evaluated in 3D in primary and secondary bone and shown as differences induced by 5, 35, or 65 days of SN (*vs*. contralateral) for multiple parameters (**Figure 6**). Please note that, as with study A, these figures do not conventionally show parameter values but instead show the differences per parameter induced by SN relative to contralateral at the three timepoints for multiple parameters (as a positive percentage difference independent of the direction of change). Furthermore, the histogram bars in **Figure 6** are colour-coded to the significance of difference.

A comparison of the separate 3D evaluation shows that, at 5 days (**Figure 6A**), all 10 parameters exhibit a significant difference in the primary region, while half were unchanged in the secondary region. With the exception of Tb.Th, all morphometric parameters showed changes in the primary region that were of higher magnitude or significance, or both, than in the secondary spongiosa relative to contralateral control at 5 days post-SN. This is especially evident for Tb.Sp, Tb.N SMI, uPi, and Conn.D which are significantly modified in primary spongiosa, but not in secondary spongiosa, after 5 days, denoting more substantial and significantly rapid shifts in morphology in the primary region due to SN.

In contrast, the quantitative differences at 35 days post-SN (**Figure 6B**) were more similar in the primary and secondary regions. The SN-induced differences at 35 days were roughly equivalent across the whole spongiosa and reached significance in both primary and secondary regions for all parameters, except Tb.Th in the former and Tb.Sp in the latter. This trend to primary–secondary equivalence was also seen 65 days post-SN (**Figure 6C**) despite significant differences in more parameters in primary *vs*. secondary bone. These data indicate that primary and secondary bones can exhibit a divergent trajectory of SN-induced morphological change depending on time post-operation.

### Study B: Spatially resolved metaphyseal profiling reveals more clearly the discrepancy in neurectomy response in primary and secondary spongiosa

**Figure 7:**
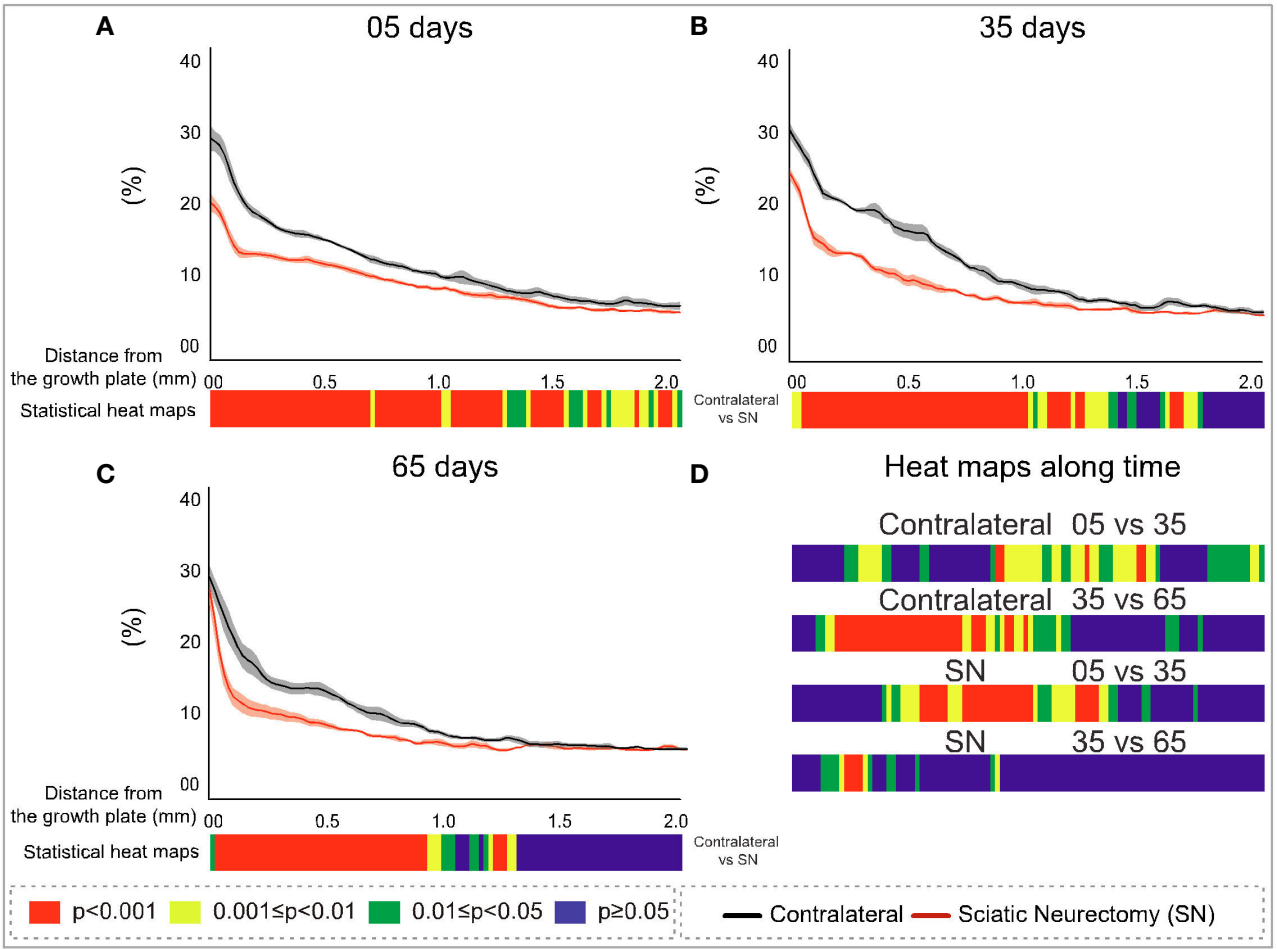
Cross-sectional (2D) percent bone area per total area (B.Ar/T.Ar) with distance from the growth plate in study B in sciatic-neurectomised (SN, red) and contralateral tibias (black) at **(A)** 5 days, **(B)** 35 days, and **(C)** 65 days after surgery. For all profiles, the SEM ranges are shown graphically. In each of the three charts, the colour bar below shows the *p-*value of the significance of difference (chi-square) by cross-sectional plane (downstream distance) between the left contralateral and right SN tibias. Additional colour bars **(D)** show if changes occurred earlier or later in the study based on the significance of difference of cross-sectional B.Ar/T.Ar with downstream distance in time-based comparisons spanning early and late time intervals for SN and contralateral control limbs.

**Figure 8:**
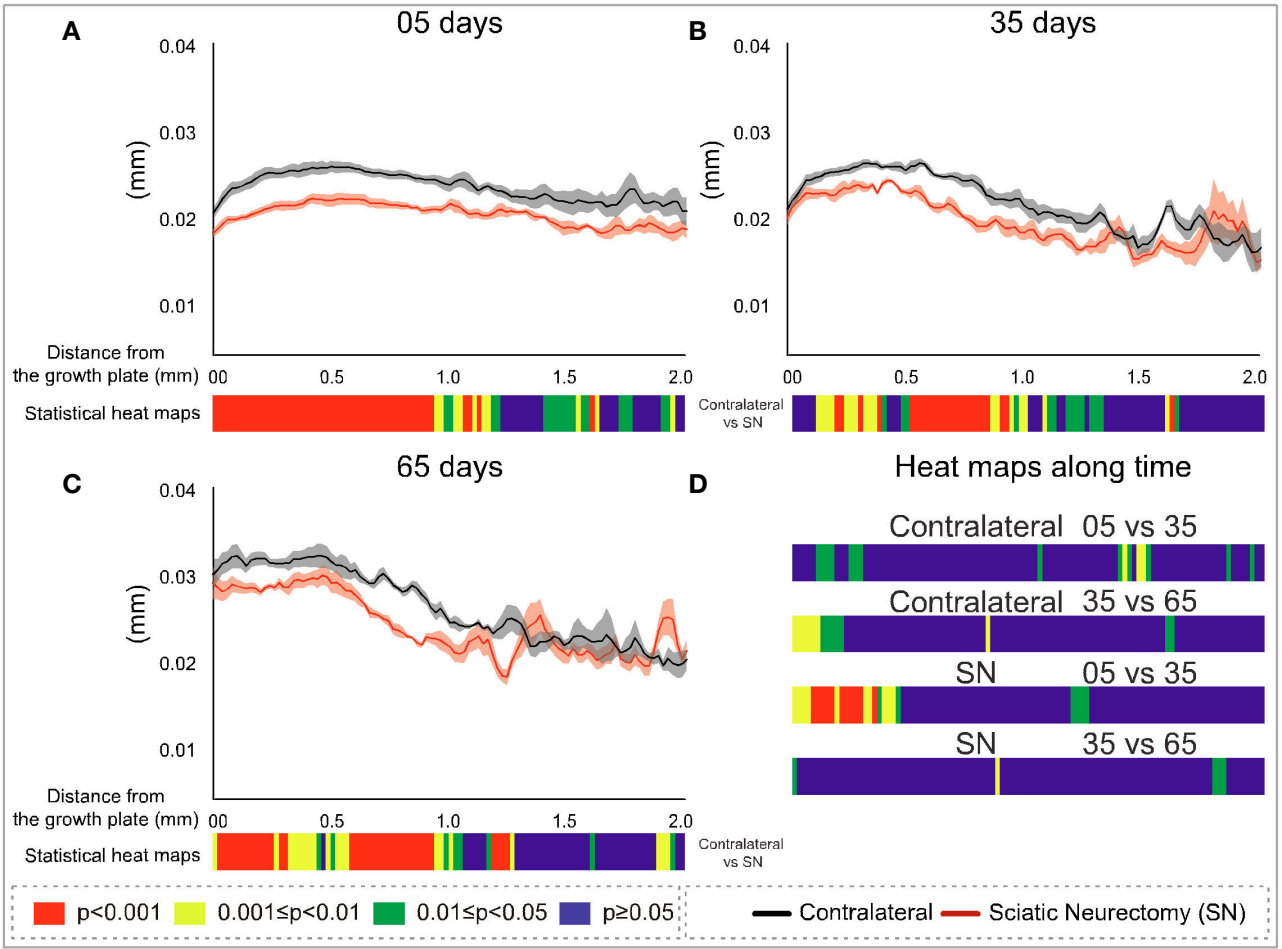
Cross-sectional (2D) trabecular thickness (Tb.Th) with distance from the growth plate in study B in sciatic-neurectomised (SN, red) and contralateral tibias (black) at **(A)** 5 days, **(B)** 35 days, and **(C)** 65 days after surgery. For all profiles, the SEM ranges are shown graphically. In each of the three charts, the colour bar below shows the *p*-value of the significance of difference (chi-square) by cross-sectional plane (downstream distance) between the left contralateral and right SN tibias. Additional colour bars **(D)** show if changes occurred earlier or later in the study based on the significance of difference of the cross-sectional Tb.Th with downstream distance in time-based comparisons spanning early and late time intervals for SN and contralateral control limbs.

**Figure 9:**
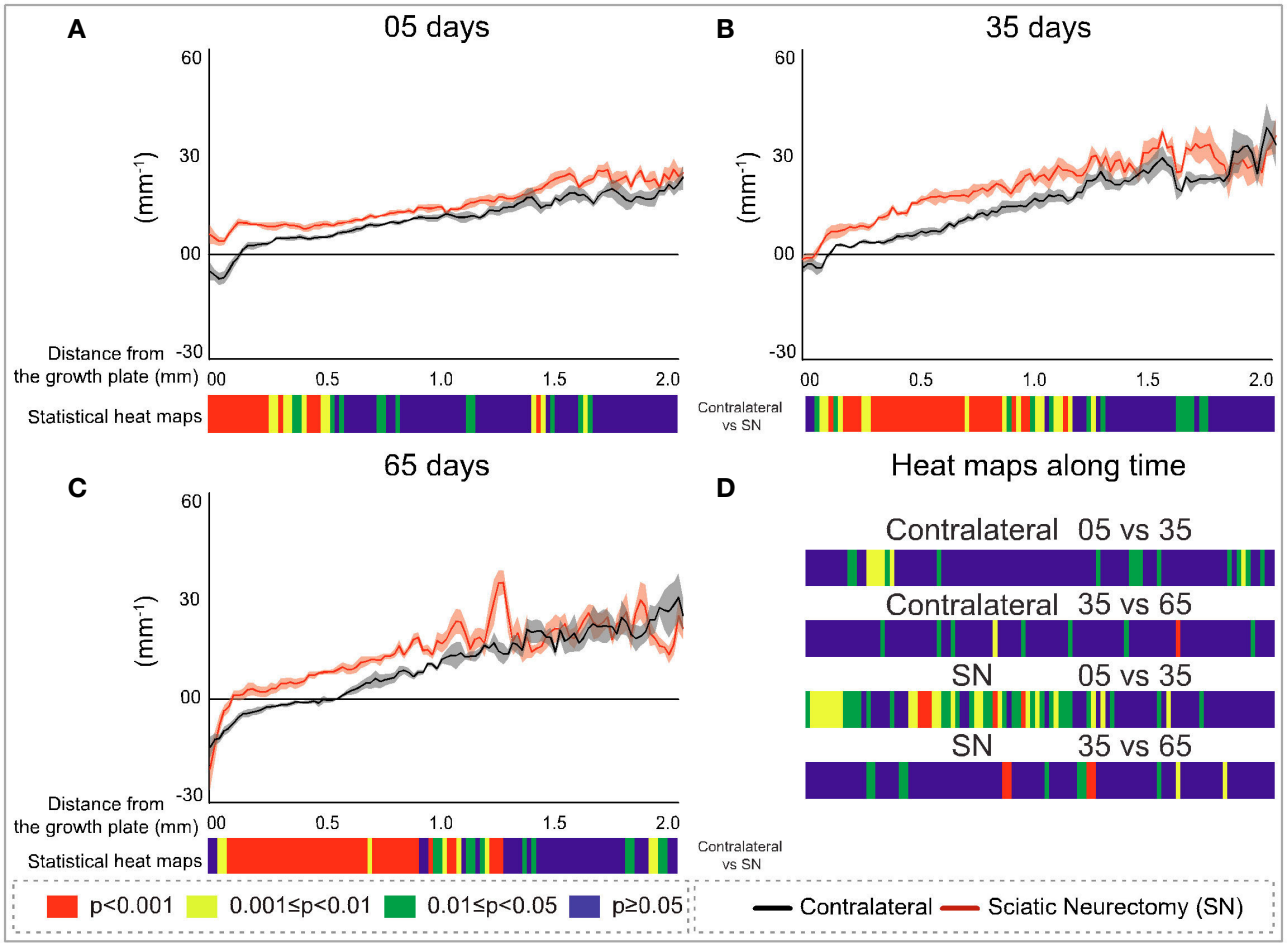
Cross-sectional (2D) trabecular bone pattern factor (Tb.Pf) with distance from the growth plate in study B in sciatic-neurectomised (SN, red) and contralateral tibias (black) at **(A)** 5 days, **(B)** 35 days, and **(C)** 65 days after surgery. For all profiles, the SEM ranges are shown graphically. In each of the three charts, the colour bar below shows the *p*-value of the significance of difference (chi-square) by cross-sectional plane (downstream distance) between the left contralateral and right SN tibias. Additional colour bars **(D)** show if changes occurred earlier or later in the study based on the significance of difference of the cross-sectional Tb.Pf with downstream distance in time-based comparisons spanning early and late time intervals for SN and contralateral control limbs.

**Figure 10:**
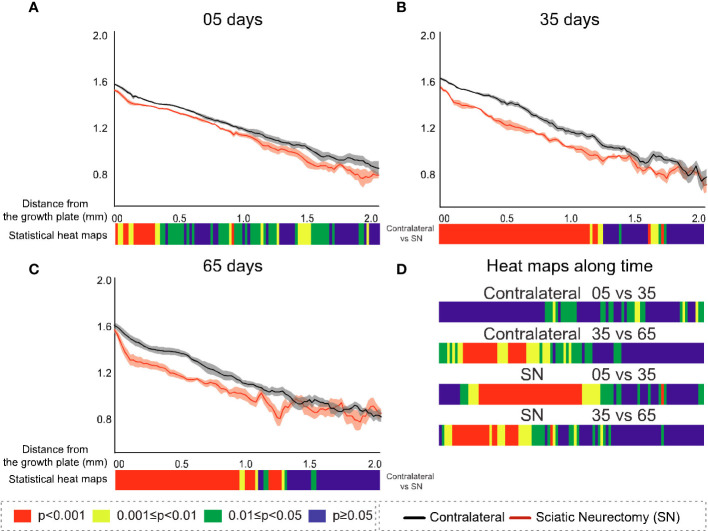
Cross-sectional (2D) fractal dimension (FD) with distance from the growth plate in study B in sciatic-neurectomised (SN, red) and contralateral tibias (black) at **(A)** 5 days, **(B)** 35 days, and **(C)** 65 days after surgery. For all profiles, the SEM ranges are shown graphically. In each of the three charts, the colour bar below shows the *p*-value of the significance of difference (chi-square) by cross-sectional plane (downstream distance) between the left contralateral and right SN tibias. Additional colour bars **(D)** show if changes occurred earlier or later in the study based on the significance of difference of the cross-sectional FD with downstream distance in time-based comparisons spanning early and late time intervals for SN and contralateral control limbs.

To further explore the divergent sensitivity to SN in primary and secondary bone, we performed profiling to spatially resolve 2D changes in B.Ar/T.Ar, Tb.Th, Tb.Pf, and FD with a specific distance from the growth plate (**Figures 7**–**10**, respectively). Pairs of profiles for each parameter at 5, 35, and 65 days are accompanied by colour bars denoting the location-matched 2D *p*-values (from chi-square analysis) for both SN *vs*. control (A–C) and spatially mapped significance of difference with time between 5–35 and 35–65 days to compare early and late changes separately for the contralateral and SN tibiae (D).

The B.Ar/T.Ar profiles show significant differences between SN and control tibiae throughout the metaphysis at 5 days that were highest in primary spongiosa (**Figure 7A**). By 35 days, significant SN–control differences extended to only ~1.5 mm downstream, and by 65 days they were even less extensive (~1.25 mm, **Figures 7B, C**). These analyses also exposed a behaviour peculiar to a primary region within only ~0.1 mm distance from the growth plate, where differences in B.Ar/T.Ar after 5 days of SN were especially large, reduced by ~half by 35 days, and almost gone by 65 days (**Figures 7A–C**, respectively). This showed a steady convergence in B.Ar/T.Ar at the growth plate end of the metaphysis during prolonged SN and was supported by heat maps comparing SN–control differences where the significance in this region, closest (<0.1 mm) to the growth plate, is reduced at later timepoints.

A scrutiny of location-matched statistical evaluation with time interval allows observations regarding B.Ar/T.Ar (**Figure 7D**). Firstly, in contralateral tibiae, early (5–35 days) changes were more significant further downstream, while late (35–65 day) changes were focused more upstream; this pattern is likely age-related. In contrast, changes in SN tibiae occurred in the central metaphyseal region from 5 to 35 days and had mostly plateaued by 35–65 days when only limited changes were evident further upstream. Secondly, the overall significance of change in SN is greater at 5–35 days, while most marked changes occur at 35–65 days in contralateral tibiae.

The metaphyseal Tb.Th profiles—like B.Ar/T.Ar—showed significance of difference that was most marked and continuous at 5 days post-SN (**Figure 8A**) and became more intermittent at 35 and 65 days post-SN (**Figures 8B, C**). At all three timepoints, a significant change in Tb.Th extended a little more than 1 mm downstream. The location-matched *p*-values for Tb.Th in control tibiae at 5–35 and 35–65 days were not marked or widespread, nor were the changes in SN tibiae—apart from a region just downstream of the growth plate between 5 and 35 days (**Figure 8D**).

In contrast to B.Ar/T.Ar whose significance of difference retreated closer to the growth plate with time post-neurectomy, for Tb.Pf and FD (**Figures 9A–C**, **10A–C**, respectively), the significance of difference extended further downstream with time. The changes exhibited early (5 day) predominance in the first 200–300 μm close to the growth plate (**Figures 9A**, **10A**) and were significant only up to 0.5 mm. With time, however, the changes showed a more widespread distribution, ‘spreading’ to more than 1 mm downstream at 35 and 65 days (**Figures 9B, C**, **10B, C**). This reveals the divergent spatial targeting of the spongiosa at various stages post-SN for quantitative (B.Ar/T.Ar) and architectural (Tb.Pf, FD) trabecular parameters.

Near the growth plate, the differences with time for Tb.Pf show a trend also seen in B.Ar/T.Ar and Tb.Th, namely, a SN–control divergence that is maximal in magnitude and significance at 5 days but which reduces in both at later times. With Tb.Pf, it is notable that the SN profile at 5 days never reaches negative values near the growth plate, as is normally the case in primary spongiosa, due to the prevalence of concave nodes in its fine-textured porous bone. This signifies a profound change in primary spongiosal architecture at early times post-SN that is reversed to a more normal morphology later.

FD, while resembling Tb.Pf in the time trend of increasing downstream extent of SN–control difference, was distinctive in two respects (**Figures 10A–C**). Firstly, at 35 and 65 days, it showed a continuous highly significant difference to ~1 mm downstream, with no loss of significance close to the growth plate as in all other parameters. In the prior three parameters (B.Ar/T.Ar, Tb.Th, and Tb.Pf), the change near the growth plate decreased with time post-SN, while for FD it was raised. Secondly, FD always showed the closest to a straight metaphyseal profile of all the studied parameters. Over the first ~1 mm downstream and excepting some gradient changes very near the growth plate, the FD profiles are very close to straight lines. This is remarkable in that the B.Ar/T.Ar, Tb.Th, and Tb.Pf show a substantial change in gradient and form over this same distance, reflecting a change from primary to secondary spongiosal architecture.

The comparisons of early (5 *vs*. 35 days) and late (35 *vs*. 65 days) interval changes showed a little trend for Tb.Pf in either interval in the control (left) tibias but a more significant change for the SN (right) tibia in the earlier interval—extending approximately 1 mm downstream (**Figure 9D**). For FD, there was again much more change in the first (5 *vs*. 35 days) interval in the SN tibias than in the control tibias, centred in the central metaphysis (**Figure 10D**). In the later time interval, both SN and control tibias showed—unlike Tb.Pf—a highly significant change extending approximately 1 mm downstream.

### Study C: Comparison of primary spongiosal depth with tibial length and mouse age

**Figure 11:**
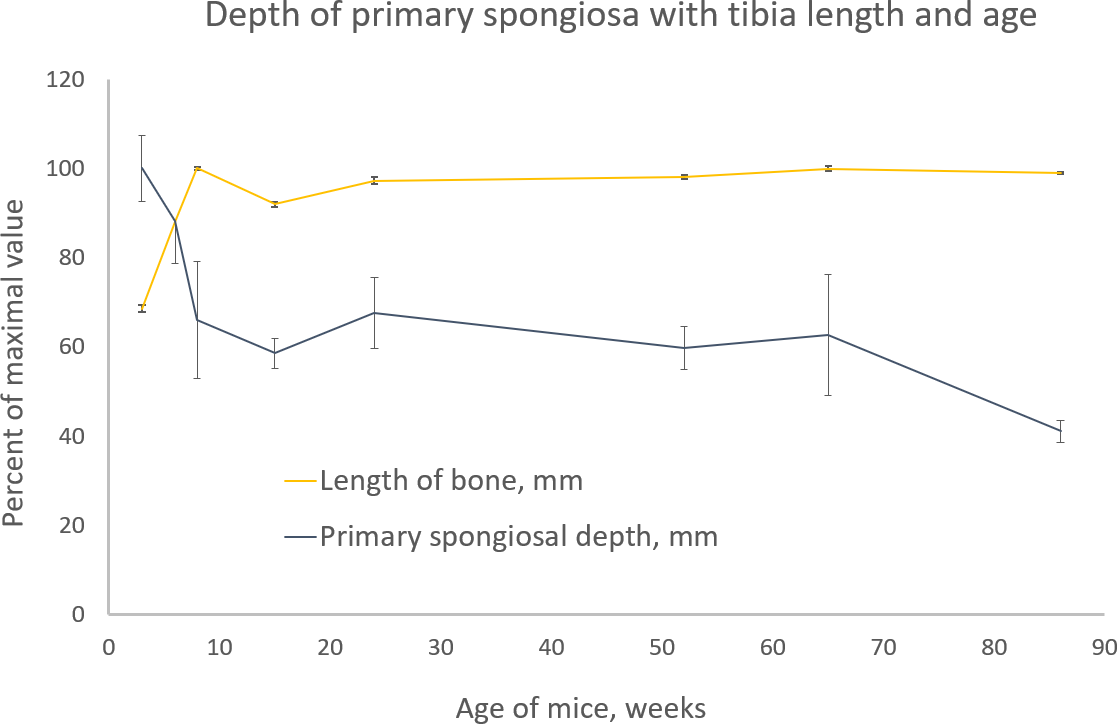
Tibia length (light orange) and primary spongiosal depth (dark blue) with the age of different groups of BL6 mice from 3 to 86 weeks. Please see text (methods) for the definition and measurement of both quantities. Both parameters are shown as percentage of the maximal value for ease of comparison. The maximal tibia length was 18.2 mm in the 8-week age group, and the maximal primary spongiosal depth was 0.5 mm in the 3-week age group.

In study C, the relationships between tibial length and primary spongiosal depth with mouse age were examined (**Figure 11**). The youngest mice were 3–8 weeks old, and in this 5-week-long interval the tibia lengthens to attain its adult extent, thereafter remaining stable throughout adulthood into very old age. Up to 8 weeks of age, the primary spongiosal depth, by contrast, decreased sharply with waning growth rate and then appeared to match tibial length at between 8 and 65 weeks (~2–15 months). At older ages than this, tibial length remained unchanged, but the primary spongiosal depth decreased by approximately a third. Thus, tibial length and primary spongiosal depth were correlated only between 8 and 65 weeks of age.

## Discussion

Adding the dimension of downstream distance has been shown herein to enhance the sensitivity and specificity of metaphyseal trabecular morphometry in experimental murine studies both of drug prevention of OVX–osteopenia and of limb disuse from neurectomy. There were certain group comparisons where changes were indeed detected in the primary region and not in the secondary region, such as the weak effect of low-dose alendronate in the OVX study and the response of the early 5-day group after SN. Therefore, analysing secondary spongiosa only, as has been customary hitherto, does not give the whole story. Moreover, the metaphyseal profiles of parameters with distance from the growth plate revealed trends and outcomes that would be missed by the unitary analysis of a spongiosal trabecular bone region.

Study A established that estradiol was fully effective, but low-dose alendronate was only partly effective, in preventing OVX-induced trabecular osteopenia in C3H mice. This difference does not reflect a fundamentally different efficacy but is instead a result of the low dose of alendronate used. Higher alendronate doses have shown a high efficacy and the full prevention of OVX-related osteopenia (35). The low-alendronate dose used in the present study, however, provided an opportunity to contrast the less efficacious treatment with the much stronger effect of high-dose estradiol. Our data show that these strong and weak effects can indeed be spatially resolved along the spongiosa with distance from the growth plate. The most significant differences were evident in the primary spongiosal region closest to the growth plate in response to low-dose alendronate treatment, with little change in regions more than 1 mm further downstream. In contrast, the stronger OVX and estradiol effects remained highly significant throughout the entire length of the metaphyseal spongiosa.

We have limited our conclusions from the morphometry data for 10 3D parameters to only the broadest rather than exploring the detailed explanations for each parameter; this acknowledges the relatively small group sizes (*n* = 4–6) and that sources of error in such studies can arise from inter-animal variation. This broad view is focused primarily on testing a null hypothesis: that the primary region near the growth plate will provide no information about trabecular bone changes caused by known anti-osteoporotic treatment or by disuse from SN. We conclude that even a conservative interpretation of the data refutes this null hypothesis emphatically. In many cases, experimentally induced differences in spongiosal architecture are strongly and significantly observed in this primary region—sometimes with greater magnitude and significance than the secondary region. It should be noted that the ‘primary region’ used in these studies corresponds to the offset region, extending from the growth plate to the downstream location where trabecular morphometry would—according to the methods mostly used to date—begin, that is, the region that would conventionally be excluded by the offset.

SN-induced disuse (study B) allowed a comparison between acute (5 day) and more chronic (35 and 65 day) trabecular changes. This revealed general trends for all parameters, with spatially resolved significance highest in the primary spongiosa close to the growth plate within 5 days of SN compared with the secondary region. In contrast, chronic changes (at 35 and 65 days) were more similar in the primary and secondary regions. More strikingly, the 2D slice-by-slice profiling showed enduring differences right up to spongiosa abutting the growth plate within 5 days of SN, whilst they diminished sharply in the region within 100–200 μm of the growth plate by 35 and 65 days (with the exception of the somewhat divergent response of fractal dimension). This suggests that bone changes induced acutely by SN involve the growth plate itself, while more chronic changes do not. This trend in temporal change in the primary spongiosa was particularly marked in Tb.Pf where—uniquely—cross-sectional values never decreased to negative values at the 5-day timepoint, while they did so (in the usual way for this type of bone) at 35 and 65 days. This gives a further indication of an acute profound change to primary spongiosa caused by SN, which appears to be reversed at later times, possibly by some limited resumption of normal formation of the primary spongiosa. The spatial mapping of temporal changes shows a general shift from downstream to upstream with time post-SN and that the shift occurs sooner in SN than in the contralateral limb (controls)—patterns that resemble a premature ageing phenotype.

Another interesting observation in the cross-sectionally resolved profiles from the SN study (B) was that a parameter of bone quantity—B.Ar/T.Ar—showed an opposite trend with downstream extent and time compared with two parameters of bone architecture—Tb.Pf and FD. For the former quantitative parameter, the significance of difference showed the greatest downstream extent at the earliest time (5 days), which become more restricted nearer to the growth plate at 35 and 65 days. In contrast, the significant architectural changes in both Tb.Pf and FD were initially, at 5 days, limited to near the growth plate but showed further downstream extent at the later timepoints. This reciprocity in the spatiotemporal pattern of bone changes in the metaphysis following SN shows that the parameters of trabecular morphological change can be nuanced and do not all simply follow percent volume.

A similar trend emerged from study A. The strong effects of both OVX and estradiol on the quantitative trabecular parameters were greater in the secondary region, while the effect on the architectural parameters (Tb.Pf and FD) was larger in the primary region. This again suggests some divergence in the evolution of trabecular morphology between the quantitative and architectural aspects.

The data from studies A and B together suggest that either weak or marginal changes or recently initiated acute changes show a higher significance in primary regions, while stronger or longer-established changes tend to manifest equally across both the primary and secondary spongiosal regions of the metaphysis. What is noteworthy by its absence is any general tendency for the significance of spongiosal changes to plummet near the growth plate. As often as not, their significance is retained and sometimes attains maximal values in this primary region. On a pragmatic basis, an analysis protocol to maximise the detection sensitivity in studies of trabecular bone might understandably extend the analysed volume of interest closer to the growth plate, reducing the offset and including some primary spongiosal bone in the analysis.

This raises the following question: is a change in primary spongiosa—either by itself or mixed with secondary spongiosa—what we are interested in discovering? Is it useful information regarding preclinical and clinical effects on bone? To date, a central paradigm of histomorphometry has been to restrict the study to secondary spongiosa only. One observation herein that might shed light on this question is the remarkably linear metaphyseal profiles for FD, which were observed in both studies A and B (OVX and SN) despite the substantial fluctuation of other bone parameters over the same spatial range. FD is important as it is an emergent property of nonlinear–chaotic pattern formation (32, 33). A linear profile for FD could thus indicate that, while trabecular architecture varies markedly downstream across the metaphysis, the underlying process of (re)modelling that is generating its emergent labyrinthine architecture (36, 37), such as a Turing (38) or Belousov–Zhabotinsky (39) nonequilibrium pattern formation process, is operating uniformly throughout the metaphysis from the growth plate to the most downstream terminus.

The linearity of the FD metaphyseal profile suggests that the process converting the primary spongiosa to secondary spongiosa acts in the same way, with the same intensity, throughout the metaphysis. Thus, although the bone’s architecture and mass will change markedly from the growth plate through to the secondary spongiosa, the underlying process of downstream change is uniform, implying that the primary spongiosa is as worthwhile a location to study bone response to exogenous intervention as any. We propose this hypothesis based upon the FD data.

It would follow that ‘primary’ and ‘secondary’ spongiosa do not exist in the strict sense as there is no categorical step-change but rather a seamless transition, without boundary. Nonetheless, a possible candidate for a pragmatic definition of this primary-to-secondary spongiosal transition is provided by the parameter Tb.Pf, a measure related to bone surface curvature and indirectly to connectivity. In the profiling of the entire trabecular region of most mice, except the most highly osteopenic, Tb.Pf starts to be negative at or near the growth plate but inflects to rising positive values downstream as the profile extends into traditionally defined secondary spongiosal regions. Negative and positive Tb.Pf values, respectively, signify a 3D morphology where the surfaces are dominated by concave and convex curvatures, respectively. Thus, the fine-textured highly-interconnected primary spongiosa with many concave connecting nodes has negative Tb.Pf, while the more widely spaced secondary spongiosa with fewer nodes and more open-ended struts will have a positive Tb.Pf. Hence, the cross-over from negative to positive Tb.Pf could provide a reference point—if one is needed—for a transition from primary to secondary spongiosal trabecular bone.

One criterion for the historical focus on the secondary trabecular bone was that of persistence—that the secondary bone will have ‘been around’ for the whole study period—making it a more valid region for analysis. The notion that no real primary-to-secondary boundary exists in terms of (re)modelling process, however, makes this requirement questionable. It is well established also that the study of bone effectively integrates all its biochemical, cellular, regulatory, and signalling components. It therefore follows that since the FD profiling points to a uniform rate of transition along the entire metaphysis, it can be proposed that even bone just recently produced at the growth plate is characteristic of the same bone regulatory regime as that found further downstream, making it valid to analyse the metaphyseal trabecular bone in its entirety. In any case, with young rodents, the metaphyseal trabecular bone can be entirely replaced—one or more times—during bone experiments of long duration, undermining the argument of bone persistence.

Another reason that the primary spongiosa may have been avoided in these analyses is that it contains more calcified cartilage from the growth plate that is remodelled into fully mineralized bone during endochondral bone growth. In mice, partial growth plate retention means that the secondary spongiosa will reduce with age, but in adult humans, where the growth plate fully mineralizes, the expectation is that, in contrast, very little primary spongiosa remains. This may have made researchers unwilling to analyse primary spongiosa in preclinical studies due to its dynamic and structural difference from the secondary spongiosa and its non-equivalence to human adult trabecular bone of relevance to osteoporosis. We are not suggesting an analysis of all primary spongiosa but only some mixed primary and secondary spongiosal regions closer to the growth plate. This aligns with the identification by Tadenev et al. of three kinds of ‘validity’ in preclinical models (40): face validity, whether they look right; construct validity, whether they arise through the right mechanism; and predictive validity, whether the results in the model will translate to humans. Our argument for including some primary spongiosa would correspond to construct validity. Thus, whilst primary spongiosa might not look right (face validity), its contribution to the data here argues against its exclusion based on any fundamental difference from the secondary spongiosa (construct validity). The metaphyseal spongiosa is a continuum, of which the primary spongiosa is just one end.

Aside from the question of offset, another recommendation arising from our studies is that downstream distance, effectively the time since formation, might be added as a dimension to metaphyseal trabecular bone analysis. The data herein have exposed differences in trabecular responses between regions close to (primary) and distant from (secondary) the growth plate. This suggests that their evaluation, separately, would allow a discrimination between acute, shorter-term, and chronic, longer-term bone responses. This can be achieved in a straightforward way using histology or microCT by the method employed by Samuels et al. (13)—and also herein—where these two distinct metaphyseal regions are examined separately. Their study (13) was indeed capable of differentiating shorter- and longer-term responses to high-dose estrogen in the tibiae of young female mice to reveal that new bone apposition occurred sooner in the upstream than in the downstream region.

Alternatively—or additionally—researchers could employ the cross-sectional 2D slice analysis method described here to yield a full resolution of the morphometric modification, with downstream distance serving as a surrogate measure of time in growing rodents. This metaphyseal profiling further enhances the information gained by morphometry by adding more resolved spatiotemporal discrimination—for example, in our studies, the metaphyseal profiles for B.Ar/T.Ar and FD showed an apparent discontinuity at ~100–200 um downstream in the tibiae subjected to SN (at both early and late timepoints; **Figures 7A–C**, **10A–C**) that was absent in contralateral tibiae. This could indicate an initially steep decline in bone formation after SN, which may align with the diminution in both B.Ar/T.Ar and FD that extends to the growth plate after 5 and 35 days, but not 65 days, post-SN. This implies that bone output from the growth plate itself was affected only at short times after SN. This also aligns with the convergence in profiles for B.Ar/T.Ar and FD by 65 days post-SN at the growth plate.

This evaluation of the temporal effects of SN is an example of the benefits afforded by metaphyseal profiling that demonstrates its utility in yielding additional mechanistic information. Based on the view that there is a normal steady transition from mostly formation at the growth plate to mostly resorption further downstream at the metaphyseal terminus, it is tempting to speculate that profile divergences near the growth plate may be related to changes in bone formation, while those further downstream may instead be more closely linked to changing resorption rates. This paradigm receives further support from the profiles of cross-sectional percent bone area (B.Ar/T.Ar) in study A as described above (**Figure 4A**). Interpreting the downstream distance of the near-growth plate profile maximum peak in this parameter as a surrogate for primary spongiosal depth, OVX greatly reduces this depth, and while alendronate treatment does not change this depth from OVX values, estradiol treatment restores it to near-sham values. To reiterate, if we consider the metaphyseal profile at its upstream end to be dominated by formation and at its downstream terminus by resorption, then estradiol, known to have bone anabolic effect at a sufficient dose, would be expected to more distinctly affect the profile near the growth plate than the antiresorptive alendronate. This is what was observed: estradiol restored the apparent depth of the primary spongiosa to near-sham value (unlike alendronate) and also caused a localised upward excursion in the B.Ar/T.Ar profile over the region within 300 μm of the growth plate, which is suggestive of a local enhancement of bone formation. It is evident, regardless, that this form of spatial-resolved morphometry, with distance from the growth plate, yields more information than a *static* image of the metaphysis.

Thus, in summary, a separate 3D analysis in primary and secondary metaphyseal spongiosa adds a temporal component to better identify trabecular changes; this analytical strength is further improved, as in cortical bone (41), by spatially resolved 2D profiling. Spatially resolved 2D metaphyseal profiling also benefits, in turn, from the reduction or elimination of an arbitrary offset, which would otherwise restrict the extent of the analysed trabecular region.

Our data indicate that trabecular bone analysis can be extended closer to the growth plate and that morphometric analysis can justifiably include more primary spongiosal bone than hitherto, without loss in sensitivity to bone changes and possibly with increased sensitivity to slight, marginal, and early changes. This may also have practical implications in ageing studies in old mice in which the metaphyseal bone is very much reduced and often appears as rare primary spongiosa and even sparser secondary spongiosal bone. Using both primary and secondary spongiosa in this case—with very little offset—may improve the quality of data from such aged model studies.

Pragmatic reasons remain, for retaining a small offset from the growth plate in selecting the region for analysis, related to the ease and accuracy of delineating trabecular from cortical bone and excluding epiphyseal trabecular bone which, due to the convoluted topography of the growth plate, extends downstream in some locations. Alternatively, this issue of the convolution of the growth plate could be addressed by conducting a morphometric analysis in the longitudinal (coronal or sagittal) rather than transaxial plane, allowing the analysis to be consistently extended closer to the growth plate while excluding the epiphyseal trabecular bone and the growth plate itself.

Finally, the data in which we have measured primary spongiosal depth and tibial length in mice of advancing age (study C) shed light on the impact of the setting of a fixed offset. We show that while tibial length is growing towards its final adult value between 3 and 8 weeks, primary spongiosal depth is anti-correlated with length during this period and reduces significantly with declining growth rate. However, at 8 weeks, longitudinal growth more or less ceases, and from then up to 15 months, the majority of the mouse’s adult life, tibial length and primary spongiosal depth show little change and more or less correlate with each other. However, after 15 months of age, the primary spongiosal depth effectively shrinks to occupy a shallower and reduced band, with no corresponding change to the tibial length. Thus, during the 8-week to 15-month interval, the practice of adjusting trabecular VOI location and extent by scaling with tibia length is supported by this data. Such scaling adjustment is not possible outside of this age range, however, when the correlation between tibial length and spongiosal depth is not retained.

## Data availability statement

The raw data supporting the conclusions of this article will be made available by the authors, without undue reservation.

## Ethics statement

The animal study A was reviewed and approved by the Animal Institutional Care and Use Committee of Galapagos controlled by the French Authorities (agreement number c93-063-06, DDPP, Seine Saint Denis). For studies B and C all procedures complied with the Animals (Scientific Procedures) Act 1986 of the UK Local Ethics Committee guidelines and were covered by HO Project license number 70/07859.

## Author contributions

All authors listed have made a substantial, direct, and intellectual contribution to the work and approved it for publication. PS and AP contributed to the conception and design of the study and wrote most of the manuscript. AP participated in all the experimental work except study A. LO performed the in vivo experimental work for study A. GK did the microCT scans for study A while PS did microCT 3D data analysis for studies A, B and C. BJ performed the in vivo experimental work and microCT scanning and analysis for study B and C. SM analysed the data, performed statistical analysis and provided the colour coded profile charts for both studies A and B.
